# Implicit memory reduced selectively for negative words with aging

**DOI:** 10.3389/fnagi.2024.1454867

**Published:** 2024-10-09

**Authors:** Sandra L. Ladd, John D. E. Gabrieli

**Affiliations:** Department of Brain and Cognitive Sciences, McGovern Institute for Brain Research, Massachusetts Institute of Technology, Cambridge, MA, United States

**Keywords:** positivity effect, implicit memory, priming, negative words, aging

## Abstract

**Background:**

Disproportionally better memory for positive versus negative information (mnemonic positivity effect, MPE) in older versus younger adults has been reported on tests of explicit memory (direct, intentional) as measured by recall and recognition. The purpose of this investigation was to examine whether the MPE would be observed for implicit memory (indirect, unintentional) under conditions where, based on previous research using single words, it was expected that the MPE for explicit memory would be absent.

**Methods:**

This study investigated the influence of age on explicit and implicit memory for positive, negative, and neutral single words as measured by yes/no recognition and word identification on 24 older adults (aged 66–85) and 24 younger adults (aged 18–37) recruited from community centers in South Boston, Massachusetts.

**Results:**

Older adults had lower recognition memory accuracy for positive, negative, and neutral words than younger adults, and, consistent with most prior studies, did not exhibit an explicit memory MPE for single words. For both groups, recognition accuracy was greatest for negative words, and was similar for positive and neutral words. In contrast, older adults exhibited implicit repetition priming, as measured by superior identification performance for repeated words, that was similar to younger adults for positive and neutral words. In younger adults, implicit memory was significantly greater for negative words than for positive and neutral words, whereas in older adults there were no significant differences in implicit memory for negative, positive, and neutral words. Therefore, selectively reduced priming for negative words in older adults was found in the context of enhanced priming for negative words in the younger adults.

**Conclusion:**

These findings show that there was an implicit memory MPE in older adults for words even under conditions where there was no explicit memory MPE in the older adults. Dampening of negative valence implicit memory with aging expands the perimeter of the age-related positivity framework.

## Introduction

The idea that aging is associated with enhanced attention to and memory for positive emotions was first described as “the positivity effect in attention and memory” by Mather and Carstensen (2003, 2005). The positivity effect is considered a well-established finding (Reed et al., 2014). However, the mechanisms that explain the effect remain uncertain (Barber and Kim, 2022). There is debate regarding the conditions under which the positivity effect is observed and whether the effect is supported more by automatic than controlled processes (Reed and Carstensen, 2012; Isaacowitz, 2022; Zsoldos and Hot, 2024).

Here we focused on the mnemonic positivity effect (MPE) in aging, namely superior memory for positive relative to negative information in older adults compared to younger adults. We examined, for the first time, whether the age-related MPE extends to implicit (indirect, unintentional) memory. This form of memory is thought to be mediated by automatic processes (Schacter, 1987, 1992). Examining the MPE for implicit memory was limited to one stimulus category, single words. Using single words was our methodological approach for the following two reasons: (a) to facilitate dissociating the effects of the MPE by memory type because the explicit memory MPE is typically absent for single words (see references below), and (b) to examine the MPE using a well-established implicit memory measure, word identification. Because valence (level of pleasantness from positive to negative) is thought to be the most important emotional feature when participants are asked to rate words (Barrett and Russell, 1999), here positive and negative words were categorized by valence ratings while equating for arousal (level of intensity from calm to excited).

Studies on the MPE for single words have often focused on explicit memory (direct, intentional) as measured by tasks that ask participants to deliberately remember a particular episode by employing the memory protocols of free recall, cued recall, and recognition. Most studies using single words have not observed an explicit memory MPE in older adults when memory was measured by *recall* (Fleming et al., 2003, control group; Grühn et al., 2005; Fernandes et al., 2008; Majerus and D’Argembeau, 2011, Experiments 2–3), *corrected recognition* (Leigland et al., 2004; Kensinger et al., 2007; Spaniol et al., 2008, Experiments 1–2), *forced-choice recognition* (Thapar and Rouder, 2009), or *recollection* using the remember-know paradigm (Kapucu et al., 2008; Piguet et al., 2008). One exception was the finding of an MPE for low-arousing, but not high-arousing, positive and negative words (Kensinger, 2008).

Here we asked if the age-associated MPE would be found for implicit memory for valenced words, even under conditions where no age-associated MPE was expected for explicit memory for words. Further, most prior studies examining memory for valenced words used norms based only on young adults, and we used positive and negative words from the most extensive norms available in the literature that included older and younger adults (Warriner et al., 2013).

To our knowledge, this is the first study on age-related performance for implicit memory with valence-specific words (positive, negative, and neutral) on healthy adults with incidental encoding (read the words) held constant and a focus on retrieval using a standard implicit memory measure (word identification). One study reported observing the positivity effect in elderly adults with both explicit and implicit measures by comparing their scores on a dispositional optimism questionnaire (Life Orientation Test) with a reaction time test designed to measure differential associations of two target concepts with an attribute (Implicit Association Test) (Panebianco et al., 2023). This study reported a positivity effect with both the explicit and implicit “measures,” but neither of these tests represent a typical assessment of explicit or implicit memory. Another study investigated the effect of aging on the processing of emotional irrelevant speech. This study compared younger and older adults on an implicit memory measure, degraded word-identification, for irrelevant speech (Van Gerven and Murphy, 2010, Study 2). However, this study did not screen participants on the standard preliminary measures used in aging and memory research (e.g., estimate of crystallized intelligence and the assessment of mild cognitive impairment or dementia). Participants were also not screened on emotional measures such as depression or anxiety. Positive, negative, and neutral Dutch words were selected from a normative data base comprised of 352 first year psychology students (age not reported) who rated words only on valence (Hermans and De Houwer, 1994). This study found implicit memory for irrelevant speech, but no interaction with word valence and age. The lack of a positivity effect reported for implicit memory with words in this study is difficult to interpret due to limitations associated with the lack of screening on cognitive and emotional measures, a normative word data base that did not include older adults, and valence-specific irrelevant words that were not rated on arousal. Valence and arousal are known to interact (Kensinger, 2009; Mather and Sutherland, 2009). Also using the auditory modality but with musical stimuli, the positivity effect was not observed for implicit memory as measured by melody preference (Narme et al., 2016). The identification of emotional facial expressions in younger and older adults was also examined using an implicit memory measure and the positivity effect was not observed (García-Rodríguez et al., 2009). However, faces may represent a special stimulus category (Tsao and Livingstone, 2008). The positivity effect has not been observed for faces across many explicit memory tasks including videos (Hayes et al., 2020). In a partial investigation of the positivity effect younger and older adults were asked to categorize negative and neutral scenes using a perceptual priming task. Studied versus novel scenes were initially presented subliminally with the exposure duration being gradually increased until the scene was accurately categorized. An age difference in emotional priming was not observed (LaBar et al., 2005). Within the meta-analysis of the age-related positivity effect (Reed et al., 2014), a reference to implicit memory was included, but the cited study did not use a standard implicit memory measure; it was an investigation of false recollection of emotional pictures (positive, negative, and neutral) in younger and older adults (Gallo et al., 2009). Also, the procedure for memory testing was not equivalent between age groups. Older adults took the memory test immediately following the study phase, while younger adults were tested 24 h later. False recollection was greater for emotionally arousing relative to neutral non-arousing pictures in both age groups, but a positivity effect was not observed.

Therefore, it is difficult to interpret these studies for multiple reasons, but especially because the measures of “implicit” memory (e.g., priming) were idiosyncratic and unrelated to measures of implicit memory that have been shown to dissociate from explicit memory. Here we used a measure of implicit memory, word-identification repetition priming, that has been shown to be preserved in older adults with reduced explicit memory relative to younger adults (Light and Singh, 1987; Hashtroudi et al., 1991; Fleischman et al., 2004) and in amnesic patients, such as the patient H.M., with severly impaired explicit memory (Cermak et al., 1985; Paller et al., 1991; Carlesimo, 1994; Paller and Mayes, 1994; Postle and Corkin, 1998). These kinds of dissociations between impaired explicit memory and intact implicit memory functions have given rise to the multiple memory systems theory: explicit and implicit memory are distinct and mediated by functionally independent cognitive and neural systems (e.g., Schacter, 1987; Gabrieli, 1989, 1998; Tulving and Schacter, 1990; Squire, 1994, 2004, 2009). Although multiple memory systems theory has been described as the prevalent paradigm in memory research for more than thirty years (Ferbinteanu, 2019), the debate with the opposing theory that priming and recognition are driven by a unitary memory system and that aging reduces both priming and recognition, even on perceptual tasks, remains unresolved, perhaps due to the use of multiple kinds of implicit measures and diverse participants across studies (Chiarello and Hoyer, 1988; Russo and Parkin, 1993; Wiggs and Martin, 1994; Small et al., 1995; Keane et al., 2004; Soldan et al., 2009; Berry et al., 2012; Ward et al., 2020; Ward, 2024).

The present study used a mixed factorial design with one between-participant factor, age (younger, older), and two within-participant factors, word valence (positive, negative, neutral) and memory type (implicit, explicit). Encoding (study phase) was identical for both memory types: healthy younger and older adults incidentally encoded the same positive, negative, and neutral words by reading them aloud. Retrieval (test phase) for implicit memory was time to perceive a word emerging from a visual mask, a form of perceptual or word identification, and for explicit memory was yes/no recognition. Implicit memory (study, test) was administered first to avoid contamination from explicit memory (study, test) which was presented second. Because older and younger adults typically have different levels of performance on word identification, we used a procedure designed to enhance its sensitivity by eliminating ceiling effects without increasing test trials (e.g., a control for fatigue effects in older adults). This procedure determined each participant’s identification threshold (IT) on masked neutral words, fine-tuned their individual IT during the practice trials on masked valence-specific words, and automatically applied the same below threshold rule adjustments to both age groups during the word identification test, an adjustment based on their individual practice trial performance. This procedure was designed to equate task difficulty between age groups, and thus eliminate the need for post-test baseline equivalence analysis as well as enhance accuracy for the dependent variable.

The valence-specific words were counterbalanced across implicit and explicit memory measures so that no participant received the same words for each memory type but across participants the same words were presented for each memory type. The incidental encoding instructions and the exposure duration of 2 s for words presented during study were both based on the largest longitudinal study comparing younger and older adults on implicit and explicit memory, a study that used word identification to test implicit memory and recall and recognition to test explicit memory (e.g., with neutral words). This study reported a dissociation between implicit and explicit memory measures for older relative to younger adults: implicit memory was spared while explicit memory was impaired (Fleischman et al., 2004).

For explicit memory, it was hypothesized that older adults would exhibit reduced recognition memory for words, and that there would not be an explicit memory MPE for words. For implicit memory, it was hypothesized that older adults would show implicit repetition priming that was comparable to younger adults. The critical question was whether older adults would show an MPE for implicit memory: disproportionately better memory for positive than negative words in older relative to younger adults. A supplementary question that has not been addressed in the literature was whether an age-related positivity bias would be observed with the perceptual identification of baseline words during the test phase regardless of implicit memory.

## 2 Materials and methods

### 2.1 Participants

Twenty-four younger adults (YA group) (*M*_age_ = 27.46 years, *SD* = 6.15; age range 18–37) and 24 older adults (OA group) (*M*_age_ = 73.67 years, *SD* = 4.86; age range 66–85) participated in this study. Each age group included 12 males (YA, *M*_age_ = 27.25 years, *SD* = 6.38; OA, *M*_age_ = 73.83 years, *SD* = 5.31) and 12 females (YA, *M*_age_ = 27.67 years, *SD* = 6.18; OA, *M*_age_ = 73.50 years, *SD* = 4.60). Written informed consent was obtained from all participants and approved by the Massachusetts Institute of Technology (MIT) Institutional Review Board (Committee on the Use of Humans as Experimental Subjects). Study participants were compensated at the rate of $20/hour.

Participants were recruited from the following community centers in South Boston, Massachusetts: Concon Community Center, Curley Community Center, South Boston Neighborhood House, and Tynan Community Center. Recruitment was based on their self-assessment as being a healthy adult as well as meeting the following criteria: (a) absence of any neurological disorders, such as cognitive decline or dementia, and associated medications; (b) absence of any psychiatric disorders, such as clinical depression or anxiety disorders, and associated medications; (c) age 18–38 or 65–85; and, (d) responded to an advertisement posted on community center billboards for research sponsored by MIT but conducted at local community centers. Adults who met these criteria were informed that if they consented to participate in this study and any of their scores on emotional inventories indicated a need for health treatment, they agreed to both receiving this information during debriefing and, also, to seeking follow-up care at a facility of their choice or from a list of free health care clinics in the Boston area that would be provided to them. Participants were excluded if they exhibited evidence of (a) mild cognitive impairment or dementia by scoring < 27 on the Mini-Mental State Examination (MMSE; Folstein et al., 1975), (b) severe depression by scoring ≥ 29 on the Beck Depression Index-II (BDI-II; Beck et al., 1996), or (c) high trait anxiety by scoring ≥ 19 on the short version of the Spielberger State–Trait Anxiety Inventory (STAI-trait form; Spielberger et al., 1983; Zsido et al., 2020). From the original recruitment sample, two participants in the OA group did not meet the MMSE inclusion criteria. In the YA group, one participant did not meet the BDI-II inclusion criteria and one participant did not meet the STAI-trait inclusion criteria.

G*Power (Version 3.1.9.7) was used to conduct an a priori power analysis (Faul et al., 2009). Based on the meta-analysis conducted by Reed et al. (2014), it was determined that a small to moderate effect size would be adequate in the current study to show a significant positivity effect (*f* = 0.19). With an alpha = 0.05, power (1-β) = 0.8, number of groups = 2, number of repeated measures = 3, and an assumed correlation among repeated measures = 0.5, a total sample of 48 (24 per YA and 24 per OA) was sufficient to power a repeated measures ANOVA with an interaction term.

### 2.2 Preliminary measures

Age group comparisons for the preliminary measures described below are presented in Table 1. Except for working memory performance, which was better for younger adults relative to older adults, age group and gender differences across these measures were not significant.

**TABLE 1:** Preliminary testing as a function of age group and gender.

	OA (*n* = 24)		YA (*n* = 24)				
Preliminary test	*M*	*SD*	*M*	*SD*	*t*	*p*	*d*
LET-NUM	7.00	3.01	10.63	2.58	4.50	0.001	1.29
AMNART	99.74	9.11	100.24	9.78	0.20	0.183	0.05
MMSE	29.17	0.87	29.29	0.75	0.53	0.596	0.15
BDI-II	5.21	6.12	3.98	4.85	0.77	0.444	0.22
STAI–TRAIT	34.46	7.14	32.67	8.06	0.82	0.419	0.24
SES	36.96	11.56	38.13	10.57	0.37	0.717	0.11
	**Male (*n* = 24)**		**Female (*n* = 24)**				
**Preliminary test**	***M***	***SD***	***M***	***SD***	***t***	***p***	***d***
LET-NUM	8.88	3.11	8.75	3.56	0.13	0.90	0.04
AMNART	100.12	8.88	99.87	9.90	0.92	0.93	0.03
MMSE	29.25	0.79	29.21	0.83	0.18	0.86	0.51
BDI-II	5.13	6.11	4.06	4.87	0.67	0.51	0.19
STAI–TRAIT	34.17	8.29	32.96	6.93	0.55	0.59	0.16
SES	37.17	10.83	37.92	11.33	0.23	0.82	0.07

OA, Older adult; YA, Younger adult; *M*, Mean; *SD*, Standard deviation; d, Cohen’s d; LET-NUM, Letter-Number Sequencing subtest from the Wechsler Adult Intelligence Scale-III; AMNART, American version of the National Reading Test; MMSE, Mini-Mental State Examination; BDI-II, Beck Depression Index-II; STAI–TRAIT, State–Trait Anxiety Inventory, Trait form short version; SES, Hollingshead Socioeconomic Status Scale.

#### 2.2.1 Working memory

The Letter-Number Sequencing subtest from the Wechsler Adult Intelligence Scale (WAIS-III; Wechsler, 2002) was used as the measure of working memory capacity (WM). Participants were read a combination of numbers and letters, and then asked to recall first the numbers in ascending order and then the letters in alphabetical order. The WM score was the maximum number of items reordered and recalled correctly. WAIS-III and its subtests have strong validity and reliability (Kaufman and Lichtenberger, 2005).

#### 2.2.2 Estimate of crystallized intelligence

The American version of the National Reading Test (AMNART; Grober et al., 1991) was used to estimate crystallized intelligence quotient (IQ) based on the equation that includes AMNART errors and years of education. The AMNART has been shown to be a valid and reliable measure (Uttl, 2002) that has utility as an estimate of crystallized IQ (Lowe and Rogers, 2011).

#### 2.2.3 Assessment of mild cognitive impairment or dementia

The Mini-Mental State Examination (MMSE; Folstein et al., 1975) was used to assess evidence of mild cognitive impairment or dementia. The MMSE has been shown to have both validity and reliability in neurologically intact elderly and is used for dementia screening (Mitrushina and Satz, 1991).

#### 2.2.4 Depression and trait anxiety

The Beck Depression Index-II (BDI-II; Beck et al., 1996), a 21-item self-report inventory, was used to measure the severity of depression in adolescents and adults. BDI-II is one of the most widely used measures in both research and clinical practice for assessing depression because of its strong validity and reliability (Osman et al., 2008; Segal et al., 2008).

The short version of the Spielberger State–Trait Anxiety Inventory was used to measure trait anxiety (STAI-trait form; Spielberger et al., 1983; Zsido et al., 2020). The STAI*-*trait form was designed to assess relatively stable individual differences in the tendency to perceive stressful situations as dangerous or threatening. Participants responded to 20 self-report questions concerning how they generally feel on a 4-point Likert scale. The validity and reliability of the STAI-trait form has been established in over 3,300 studies, including research in medicine, dentistry, education, and the behavioral sciences (Spielberger et al., 1983; Kaplan and Saccuzzo, 1997).

#### 2.2.5 Socioeconomic status

The Socioeconomic Status Scale (SES; Hollingshead, 1957) was used to measure social and economic status. The SES scale separately ranks an individual’s educational and occupational attainment on scales ranging from 1 to 7. A weighted score was computed by multiplying the educational score by 4 and the occupational score by 7 and summing the 2 scores. The SES scores for the older adults were compared to the parents of the younger adults, because most younger adults had not, yet, completed their educations. The validity and reliability of the SES scale has been established across a variety of different settings and samples (Cirino et al., 2002).

### 2.3 Memory measures

Below are the measures used in the experimental protocol. The details of their application for this study are given in the procedure.

#### 2.3.1 Word identification test

A word identification test was used to measure implicit memory. For the word identification test, participants were very briefly shown primed (studied) and baseline (unstudied) words one at a time as the word emerged from a visual mask. Their task was to identify the word by saying it aloud. They were required to guess on each presentation until they correctly identified the word. Following an incorrect identification, the exposure duration of the same word was increased by 16.7 milliseconds (ms). Following a correct identification, the experimenter advanced to the next word (trial). The task was similar to that used by Fleischman et al., 2004 with the following exceptions: (a) the duration of the initial presentation of the primed or baseline word was determined by the individual’s identification threshold based on neutral words and then adjusted during the practice trials that included positive, negative, and neutral words, (b) there was no maximum attempt (cut-off point) to identify each word, and (c) the word was both proceded and followed by a mask that was three symbols longer than the length of the word. There were two dependent measures: the mean exposure time required to correctly identify (a) primed (studied) and (b) baseline (unstudied) words. Priming was measured as the exposure time required to correctly identify baseline words minus the exposure time required to correctly identify primed words. Exposure times were measured in milliseconds (ms).

#### 2.3.2 Yes/No recognition test

A yes/no recognition test was used to measure explicit memory. Participants were shown target (studied) and foil (unstudied) words one at a time and asked to say “yes” to the target words (Hits) and “no” to the foils (False Alarms, FA). There were two dependent measures: the mean percentage of (a) “yes” responses to words on the study list (Hits) and (b) the mean percentage of “yes” responses to words that were not on the study list (FA). Recognition was measured as the percentage of Hits minus FA.

### 2.4 Apparatus and software

A MacBook Pro (Retina, 13-inch) laptop using macOS Big Sur (Version 11.7.4) was used to run the experiment using SuperLab Pro (Version 6) software^1^. The software was customized to (a) present protocol instructions along with each study and test phase of the implicit and explicit memory tasks in accordance with the 8 series designed for counterbalancing, (b) display study and test phase stimuli, (c) implement the Identification Threshold (IT) procedure and fine-tuning adjustment during the practice test trials for implicit memory, and (d) register the experimenter’s input of participant oral responses. To register participant responses, a USB Numeric Keypad (Insignia, Model No.: NS-PNK6A01) was used by the experimenter.^1^

### 2.5 Stimuli

#### 2.5.1 Study and test lists

A total of 216 words were selected from 13,915 English lemmas (Warriner et al., 2013). The word dimensions abstracted from this normative data and used to quantitatively balance word lists were number of letters, valence (ranging from pleasant to unpleasant), arousal (ranging from calm to excited), and frequency. The word lists used here excluded words that produced significant gender differences (e.g., weapons) or very high arousal ratings (e.g., taboo, sexual, and swear words). Words denoting disease were used within the negative word lists because their inclusion did not interfere with balancing word lists for frequency and arousal across valence categories or by gender ratings.

The 216 words were assigned to four lists of 54. The four lists served as study stimuli (A, B, C, D) and are presented in Supplementary Table 1. The study lists were constructed to equate for number of letters, frequency, and arousal (positive and negative words only) but to differ significantly on valence (positive, negative, and neutral). Means (*Ms*) and standard deviations (*SDs*) for word dimensions are presented in Table 2. Following word list construction, the study lists were initially randomized in three blocks of 18 words. Each block contained an equal number of positive, negative, and neutral words. Within each study list, the items were then re-arranged in pseudorandom order with the constraint that no more than two words from the same valence appeared in a row.

**TABLE 2:** Means and standard deviations for word dimensions.

	Words						
		Positive		Negative		Neutral	
Dimensions	*N*	*M*	*SD*	*M*	*SD*	*M*	*SD*
Frequency	72	1845.88	3174.71	1816.81	3049.04	1873.78	3305.20
Valence	72	7.78^c^	0.08	2.31^a^	0.43	4.77^b^	0.46
Arousal	72	5.12^b^	0.89	5.10^b^	0.76	3.68^a^	0.63

*M*, Mean; *SD*, Standard deviation. These word dimensions were all derived from the database developed by Warriner et al. (2013). Means with significantly different means using Tukey’s *post-hoc* tests have different superscripts (*p* < 0.001). No significant differences were found between frequency of the three types of words, *F*(2, 213) = 0.01, *p* = 0.994. For valence, significant differences were found between types of words, *F*(2, 213) = 3478.14, *p* < 0.001. Means with significantly different means using Tukey’s *post-hoc* tests have different superscripts (*p* < 0.001). For arousal, significant differences were found between word types, *F*(2, 213) = 83.86, *p* < 0.001. Means with significantly different means using Tukey’s post hoc test have different superscripts (*p* < 0.001). Notably, no significant difference was found between arousal of positive and negative words (*p* = 0.972).

The test lists were constructed by combining two study lists (AB, CD). The test lists, 108 words in each list, were initially randomized in six blocks of 18 words with each block containing an equal number of positive, negative, and neutral words. Within each test list, the items were then re-arranged in pseudorandom order (two forms for each test list) with the constraint that no more than three words from the same study list and two words from the same valence category appeared in a row.

#### 2.5.2 Identification threshold determination

To determine the identification threshold (IT), a 30-word list was constructed. All words were in the neutral valence range (rating of 5.0) with an average frequency of 1800. Neutral words were used because balancing the IT word list by positive and negative valence as well as frequency was precluded due to the limitations of the normative word data (Warriner et al., 2013). Words were randomized in blocks of 5 by the number of letters (4, 5, 6, 7, and 8). The font type and size as well as the mask was identical to that used for the word identification test lists. None of the words appeared on study or test lists.

#### 2.5.3 Fine tuning of the identification threshold during the practice trials

Fine tuning of the IT was accomplished during the practice trials that used 7 words (2 positive, 2 negative, and 3 neutral). The practice words conformed to the same number of letters, valence, frequency, and arousal parameters as that used in the construction of the test lists.

#### 2.5.4 Mask

A mask was used during the IT procedure and for the practice and word identification test trials. The mask was constructed by combining the following symbols: @%$$$$$$@%. A fixed combination of symbols, @%, framed the mask. The symbol for a dollar ($) varied and was determined by the length of the word: three-dollar symbols longer than the number of letters in the word.

### 2.6 Procedure

After signing informed consent, participants completed the preliminary tests in the following order: WAIS-III, AMNART, MMSE, BDI-II, STAI-trait form, and the SES. Based on their scores, participants were sent study invitations until comparable sample sizes across strata were reached. Invited participants were then assigned to one of eight protocol series. Using a within-participant design, the protocol series was constructed to counterbalance stimulus materials across implicit and explicit memory measures (Table 3) with the constraint that implicit memory as measured by word identification was tested before explicit memory as measured by yes/no recognition.

**TABLE 3:** Counterbalancing protocol for implicit and explicit memory.

		Word Identification			Recognition		
P	S	SL1	TL1	F	SL1	TL1	F
OA-1	1	A	AB	1	D	CD	1
OA-2	2	B	AB	1	C	CD	1
OA-3	3	C	CD	1	B	AB	1
OA-4	4	D	CD	1	A	AB	1
OA-5	5	A	AB	2	D	CD	2
OA-6	6	B	AB	2	C	CD	2
OA-7	7	C	CD	2	B	AB	2
OA-8	8	D	CD	2	A	AB	2
YA-1	1	A	AB	1	D	CD	1
YA-2	2	B	AB	1	C	CD	1
YA-3	3	C	CD	1	B	AB	1
YA-4	4	D	CD	1	A	AB	1
YA-5	5	A	AB	2	D	CD	2
YA-6	6	B	AB	2	C	CD	2
YA-7	7	C	CD	2	B	AB	2
YA-8	8	D	CD	2	A	AB	2

P, Participant; S, Series; SL, Study list; TL, Test list; F, Form; OA, Older adult; YA, Younger adult.

#### 2.6.1 Implicit memory

##### 2.6.1.1 Study phase

Participants were told that the purpose of this task was to test their ability to read words. They were given 6 practice trials composed of positive, negative, and neutral words. On each trial a central fixation cross initially appeared on the computer screen for 500 ms. A blank screen then appeared for 500 ms followed by the target word, which appeared at the fixation point and remained on the screen for 2 seconds (s). After the participant read aloud the word, the software advanced to the next trial following a 500 ms inter-trial-interval.

##### 2.6.1.2 Identification threshold (IT) and fine-tuning adjustment phase

Prior to the word identification test, each participants’ IT was determined and fine-tuned (Tables 4, 5). The IT fine-tuning rules were automatically applied to the test based on each participants’ individualized performance during the practice trials for the word identification test (correct and incorrect identifications). This new method eliminated the ceiling effect employed in previous research (e.g., stopping the task after a predetermine trial limit was reached). The last trial for each individual was based on a correct identification.

**TABLE 4:** Example of identification threshold (IT) method for the word identification test.

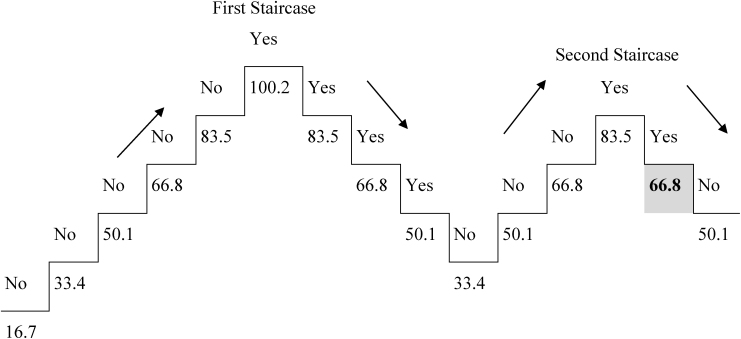			
Category	Stimulus Example	Duration (ms)	Response
Fixation	+	500	none
Mask	@%$$$$$$$@%	100	none
Word	radar	variable by staircase	none
Mask	@%$$$$$$$@%	unlimited	Y or N

In this example, the participant saw the fixation cross, the forward mask, the word for 16.7 ms, and the backward mask which remained on the screen until the participant responded with “yes” they saw a word or “no” they did not see the word. The exposure duration for each presentation of the word increased until they said “yes” and decreased until they said “no” by intervals of 16.7 ms. The staircase then repeated a second time with the final “yes” response in the second staircase being the IT time (shown in the gray box in the figure above). In this example, the IT was determined to be 66.8 ms and was used during the 7 practice trials for the test phase. Bold values indicate the exposure duration when the participant gave the correct identification for the word.

**TABLE 5:** Example of fine-tuning adjustment of the IT in practice trials for the word identification test.

Category	Stimulus Example	Duration (ms)	Response
Fixation	+	500	none
Mask	@%$$$$$$$@%	100	none
Word	wish	**66.8**	none
Mask	@%$$$$$$$@%	unlimited	Y or N

The initial IT was determined on neutral words using the staircase method (Table 4). Then practice trials were conducted prior to the test. The practice trials had two purposes: first, to acquaint the participant with the test procedure, which was different than the staircase method used to determine the IT, and second, to fine-tune the IT. In the practice trials, there were 7 words (2 positive, 2 negative, 3 neutral) presented at the participant’s IT (which was 66.8 ms in this example). The following IT fine-tuning rules were applied to the test based on their performance in the practice trials: (1) For correct responses: decrease the exposure duration (e.g., faster) by 3 levels for correct responses on 7 out of 7 trials, 2 levels for correct responses on 6 out of 7 trials, and 1 level for correct responses on 3 out of 7 trials. (2) For incorrect responses: increase the exposure duration (e.g., slower) by 3 levels for incorrect responses on 0 or 1 out of 7 trials, 2 levels for incorrect responses on 2 out of 7 trials, 1 level for incorrect responses on 3 out of 7 trials. (3) Each level was 16.7 ms. In this example, if the participant responded correctly to 7 words when presented at their IT (e.g., 66.8 ms) then during the test, the IT would be decreased by 3 levels (50.1 ms). In the test, the IT for this participant would be 66.8. An exposure duration of 50.1 = 16.7 ms would be the lowest possible IT. Bold values indicate the exposure duration when the participant gave the correct identification for the word.

##### 2.6.1.3 Test phase

Implicit memory was measured by the word identification test. Participants were told that this was a test of their visual ability. Before beginning the test, participants were given 7 practice test trials composed of two positive, two negative, and three neutral words. Their task was to identify single words that were presented very briefly on the computer screen. For each presentation, a central fixation cross appeared for 500 ms, followed by a blank screen for 500 ms. They were told to focus on the fixation cross, their signal to get ready, and that a mask would appear before and after the word. An example of the mask was given to them. After the word was presented, participants were told that the mask would stay on the screen until they attempted to identify the word. Participants were required to respond on every presentation of the word (e.g., guess) even if they were uncertain of the accuracy of their identification. The experimenter entered an N or Y on the numeric keypad to record incorrect or correct identifications. If the identification was incorrect (N), the same word would automatically appear on the screen for a longer time interval and the participant would again attempt to identify it. If the identification was correct (Y), the next word would automatically be advanced and presented very briefly starting at the fine-tuned duration based on that participant’s IT adjustment and increasing on each presentation by 16.7 ms until the word was correctly identified. When a Y response was recorded, the next trial (new word) was automatically presented following a 500 ms inter-trial-interval.

#### 2.6.2 Explicit memory

##### 2.6.2.1 Study phase

The procedure was the same as that used for implicit memory.

##### 2.6.2.2 Test phase

The participants were told that this was a test of their MEMORY for the words that they just read. Before beginning the test, the participants were given 7 practice trials composed of two positive, two negative, and three neutral words. For the recognition test, a word was presented that remained on the monitor until the participant said either “yes” if they remembered the word from the study phase or “no” if they did not remember the word from the study phase. The experimenter recorded the participant’s response which automatically initiated the next trial.

#### 2.6.3 Statistical analyses

Statistical analyses were performed using IBM SPSS Statistics (Statistical Package for the Social Sciences, version 27, SPSS Inc., Chicago, Illinois, United States). For all analyses, a significance level of *p* < 0.05 was set. For difference scores, data are depicted as mean (*M*) ± standard error of the mean (*SEM*). For single scores, data are depicted as mean (*M*) ± standard deviation (*SD*).

For implicit memory as measured by word identification, the exposure time (ms) observed to correctly identify new (baseline, unstudied) minus old (primed, studied) words was used to measure the magnitude of implicit memory (priming effect). Because it takes a longer exposure duration to identify new relative to old words, a greater difference score (new - old) indicates more priming. Implicit memory was measured by the priming effect (new - old) by valence category (positive, negative, or neutral words). To determine if there was a positivity bias in perceptual identification for baseline (new) words independent of memory, a supplementary analysis was done for valence by age group on exposure duration to correctly identify new baseline words during the study phase.

For explicit memory as measured by yes/no recognition, the count for “yes” responses to old (studied, Hits) minus new (unstudied or false alarms, FA) words was converted to percentages and used to assess the magnitude of recognition. In other words, corrected recognition scores were calculated by subtracting the FA rate, indicating a word as old when new, from the Hit rate, indicating a word as old when old. A larger difference score (old - new) indicates greater recognition, more yes responses to old relative to new words. Explicit memory was measured by the magnitude of recognition (old - new) by valence category (positive, negative, or neutral words).

The analysis makes a distinction between both implicit and explicit memory measures, computed as difference scores, and the components of each form of memory computed separately (item type: word identification for new and old words; recognition for old and new words).

## 3 Results

### 3.1 Implicit memory by age group and valence

Implicit memory was measured by priming, computed as a difference score (new - old, ms), on the word identification test. To compare the effects of age group and valence on priming, the exposure time to identify baseline (new, unstudied) minus primed (old, studied) words was entered into a 2 (age group: older vs. younger adults, between participants) x 3 (priming, within participants, by valence: positive, negative, neutral) mixed effects ANOVA. Significant differences were obtained for the main effect of priming *F*(2, 92) = 2.41, *p* = 0.05, partial η_p_^2^ = 0.05, and for the interaction between priming and age group, *F*(2, 92) = 2.40, *p* = 0.05, partial η_p_^2^ = 0.05. There was no significant difference for the main effect of age group, *F*(1, 46) = 1.26, *p* = 0.13, partial η_p_^2^ = 0.03. Independent sample *t* tests revealed that priming for negative words was lower for older adults compared to younger adults, *t*(46) = 2.03, *p* = 0.05, *d* = 0.59, or from an alternative perspective, priming for negative words was higher for younger adults relative to older adults, while no significant age difference was observed for positive or neutral words, respectively: *t*(46) = 0.09, *p* = 0.93, *d* = 0.03; *t*(46) = 0.05, *p* = 0.96, *d* = 0.02.

**FIGURE 1:**
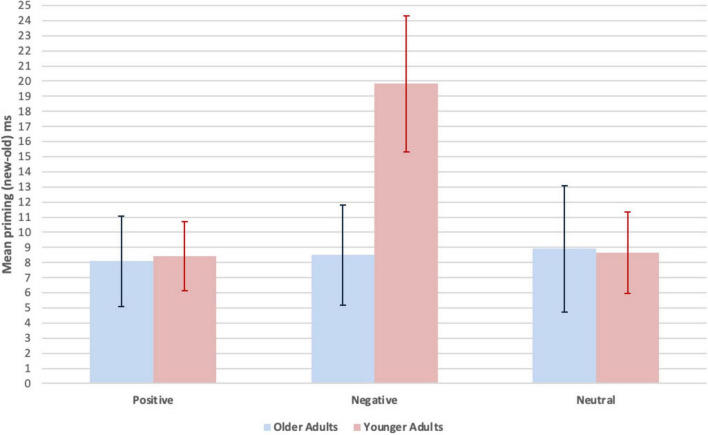
Mean priming (new-old, ms) as a function of age group and valence. Implicit memory was measured by priming. The formula was the exposure time (ms) to identify new words (unstudied) minus exposure time to identify old words (studied). Because it takes a longer exposure duration to identify new relative to old words, a greater difference score (new-old) indicates more priming. Error bars represent *M ± SEM*.

Paired samples *t* tests within the older adults showed that there were no significant differences between priming for positive and negative words: *t*(23) = 0.10, *p* = 0.92, *d* = 0.02, positive and neutral words: *t*(23) = 0.24, *p* = 0.41, *d* = 0.05, or negative and neutral words: *t*(23) = 0.07, *p* = 0.47, *d* = 0.02. Within the younger adults, priming for negative words was significantly greater than for positive words: *t*(23) = 2.98, *p* = 0.01, *d* = 0.61 and neutral words: *t*(23) = 2.48, *p* = 0.01, *d* = 0.51. There was no significant difference between priming for positive and neutral words: *t*(23) = 0.08, *p* = 0.47, *d* = 0.02. *Ms* and *SEM* for priming (new - old, ms) as a function of age group and valence are shown in Figure 1. Statistical analyses are summarized in Supplementary Table 2.

With the word identification task, verifying that priming was induced was made by comparing item type, new (unstudied) versus old (studied), for all conditions: OA – positive, negative, and neutral words; YA – positive, negative, and neutral words. A significant difference (one-tailed paired *t* tests) between these two item types for priming was obtained for all conditions and is depicted in Supplementary Figure 1. Statistical analyses are summarized in Supplementary Table 3. Proof of concept for this approach in memory research was provided by computing priming (new - old) to 0 using one-sampled *t* tests (one-tailed) for each condition. For the OA positive words, t(23) = 11.92, *p* < 0.001, *d* = 2.43; negative words, t(23) = 11.33, *p* < 0.001, *d* = 2.31; neutral words, t(23) = 8.68, *p* < 0.001, *d* = 1.77. For YA positive words, t(23) = 14.88, *p* < 0.001, *d* = 3.04; negative words, t(23) = 17.68, *p* < 0.001, *d* = 3.61; neutral words, t(23) = 14.43, *p* < 0.001, *d* = 2.95. These results confirmed the item type approach used here by demonstrating that priming occurred for all valences in both younger and older adults.

### 3.2 Perceptual identification as assessed by exposure duration to correctly identify new baseline words during the test phase

To assess whether there was a difference by age group for the time required (ms) to correctly identify baseline items in relation to valence, the exposure duration for correct identification of baseline items was entered into a 2 (age group: older vs. younger adults, between participants) × 3 (valence, within participants: positive, negative, neutral) mixed effects ANOVA. No significant differences were obtained for the main effect of age group, *F*(1, 46) = 0.001, *p* = 0.98, partial ηp^2^ < 0.01, such that younger adults (*M* = 962.18, *SEM* = 55.11) obtained a similar exposure duration for the correct identification of baseline items when compared to older adults (*M* = 964.03, *SD* = 55.10). There was a significant main effect for exposure duration by valence, *F*(2, 92) = 4.49, *p* = 0.01, partial ηp^2^ = 0.09, but no significant interaction between age group and exposure duration by valence, *F*(2, 92) = 2.58, *p* = 0.08, partial ηp^2^ = 0.05.

Paired *t* tests computed for older adults showed that the exposure duration for correct identification of baseline items was similar for positive (*M* = 937.85; *SD* = 212.78) and negative (*M* = 956.43; *SD* = 211.58) baseline words: *t*(23) = 0.49, *p* = 0.63, *d* = 0.10, and for positive and neutral (*M* = 997.81; *SD* = 247.28) baseline words: *t*(23) = 1.58, *p* = 0.13, *d* = 0.32. For older adults, there was no significant difference in the exposure duration for the correct identification of baseline items between negative and neutral words: *t*(23) = 1.31, *p* = 0.20, *d* = 0.27. For younger adults, the exposure duration for the correct identification of baseline items was significantly greater for negative (*M* = 1015.37; *SD* = 387.09) than positive (*M* = 908.29; *SD* = 315.32) baseline words, *t*(23) = 3.70, *p* < 0.001, *d* = 0.76, and neutral (*M* = 962.88; *SD* = 297.60) than positive baseline words: *t*(23) = 2.10, *p* = 0.05, *d* = 0.43. For younger adults, there was no significant difference between the exposure durations for the correct identification of baseline items for negative and neutral baseline words: *t*(23) = 1.60, *p* = 0.12, *d* = 0.33. This supplementary analysis done for valence by age group on exposure duration to correctly identify new baseline words during the test phase revealed that there was no difference in the perceptual identification of baseline (new) words independent of memory.

### 3.3 Explicit memory by age group and valence

**FIGURE 2:**
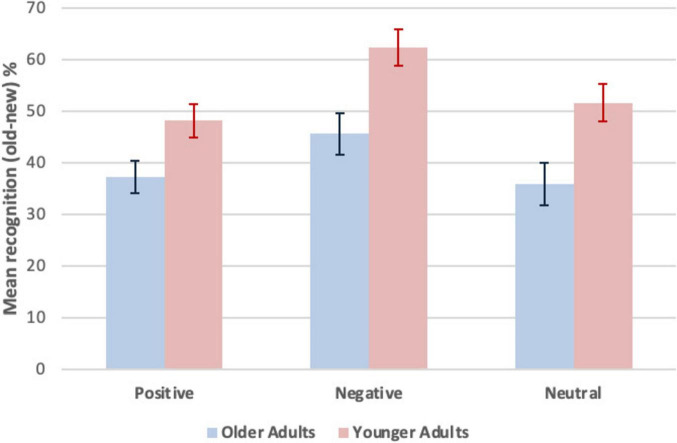
Mean recognition (old-new, %) as a function of age group and valence. Explicit memory was measured by yes/no recognition. The formula was the count for “yes” responses to old (studied, Hits) minus new (unstudied, False Alarms) words converted to percentages. A larger difference score (old-new) indicates greater yes/no recognition, more yes responses to old relative to new words. Error bars represent *M ± SEM*.

Explicit memory was measured by yes/no recognition, computed as a difference score (old - new words, %). To compare recognition by age group and valence, raw scores were converted to percentages for old - new words and entered into a 2 (age group: older adults vs. younger adults, between participants) x 3 (recognition, within participants, by valence: positive, negative, neutral) mixed effects ANOVA. Significant differences were obtained for the main effect of recognition *F*(2, 92) = 12.57, *p* < 0.001, partial η_p_^2^ = 0.22. There was no significant interaction between recognition and age group, *F*(2, 92) = 0.79, *p* = 0.23, partial η_p_^2^ = 0.02. There was a significant main effect of age group *F*(1, 46) = 11.50, *p* < 0.001, partial η_p_^2^ = 0.20, with better performance by the younger adults (*M* = 54.01, *SEM* = 3.01) than the older adults (*M* = 39.58, *SEM* = 3.01). There was a main effect of valence, with significantly better performance on negative words than either positive words, *t*(47) = 4.29, *p* < 0.001, *d* = 0.62, or neutral words, *t*(47) = 4.69, *p* < 0.001, *d* = 0.68. There was no significant difference between positive and neutral words, *t*(47) = 0.40, *p* = 0.69, *d* = 0.62. *Ms* and *SEMs* for yes/no recognition (old - new, %) as a function of age group and valence are shown in Figure 2. Statistical analyses are summarized in Supplementary Table 4.

With the yes/no recognition task, verifying that recognition was induced was made by comparing item type, old (studied) versus new (unstudied) for all conditions: OA−positive, negative, and neutral words; YA – positive, negative, and neutral words. A significant difference (one-tailed paired *t* tests) between these two item types for recognition was obtained for all conditions and is depicted in Supplementary Figure 2. Statistical analyses are summarized in Supplementary Table 5. These analyses show that explicit memory was induced in all conditions.

## 4 Discussion

To our knowledge, this is the first study to show that older adults have selectively reduced implicit memory for negative information (words) relative to younger adults with a paradigm (word identification) known to reflect implicit memory processes as documented by dissociations between explicit and implicit memory in healthy adults and in amnesic patients using this same task. Explicit memory as measured by yes/no recognition was reduced in older adults when compared to younger adults for negative, positive, and neutral words. For explicit memory, the mnemonic positivity effect (MPE), the tendency to disproportionally remember positive versus negative information in older adults, was not observed, a finding expected based on previous research using single words. Recognition accuracy was greatest for negative words in both age groups and was similar for positive and neutral words within both age groups. In contrast, implicit memory as measure by word identification, was similar in older and younger adults for positive and neutral words. Critically, an implicit memory MPE was observed because older adults exhibited implicit memory equivalent to younger adults for positive words, but older adults exhibited reduced implicit memory for negative words relative to younger adults. This finding extends, for the first time, a *version* of the MPE to implicit memory.

### 4.1 Age differences in explicit memory across all valences

Reduced recognition memory for words in the older adults is consistent with a meta-analysis of age-related effects on recognition memory (Fraundorf et al., 2019). The expected finding of no explicit memory MPE is also consistent with most previous research using explicit memory measures with positive and negative single words (Fleming et al., 2003, control group; Leigland et al., 2004; Grühn et al., 2005; Kensinger et al., 2007; Fernandes et al., 2008; Kapucu et al., 2008; Piguet et al., 2008; Spaniol et al., 2008, Experiments 1–2; Thapar and Rouder, 2009; Majerus and D’Argembeau, 2011, Experiments 2–3; an exception was Hamilton and Allard, 2020, a study that did not control for word frequency). With respect to arousal, one study reported an explicit memory MPE only for low arousing, but not high arousing, positive and negative words (Kensinger, 2008). The mean level of low arousal used here approximated that of Kensinger, but no explicit memory MPE was observed in the present study. Studies that observe the explicit memory MPE encourage naturalistic and unconstrained processing of stimuli during encoding (Charles et al., 2003; Kwon et al., 2009). The explicit memory MPE is not observed when encoding requires participants to explicitly remember all information (Grühn et al., 2005; Majerus and D’Argembeau, 2011) or is constrained. The minimal incidental encoding requirement used here was effective in producing the absence of an explicit memory MPE.

One major challenge to the age-related and valence-specific postulate described in the positivity effect remains the difficulty in equating positive and negative stimuli for arousal. This challenge was mitigated by using word stimuli because valence may play a stronger role than arousal when the valence-specific stimuli are based on the rating of words (Barrett and Russell, 1999). In addition, the positive and negative words used were equated for arousal based on the most extensive word-valence-frequency norms for younger and older adults that were available in the literature (Warriner et al., 2013).

### 4.2 Age similarities in implicit memory with a selective difference for negative valence

The novel finding reported here is that older adults showed reduced implicit repetition priming for negative words relative to younger adults, but implicit repetition priming that was similar in younger adults for positive and neutral words. There was an effect of valence on implicit memory in younger adults only, with greater priming for negative than positive or neutral words; this difference was not significant in older adults. These findings are consistent with the MPE in older adults, but are the first example of the MPE extending to implicit memory in older adults.

It is noteworthy that the age-associated difference in priming occurred in the context of greater priming only in the younger adults for negative words than for either positive or neutral words. For younger adults, the explicit and implicit measures of memory paralleled one another with greatest memory for negative words. For older adults, there was, by comparison, a relative decrease in implicit memory for negative words. Although negative words often have greater arousal value than positive and neutral words, that cannot explain the current findings because words were matched for arousal across valence categories. Further research will be needed to understand why such negative words enhanced both explicit and implicit memory in younger adults, but only explicit memory in older adults. Only the older adults exhibited a dissociation between explicit memory for negative words (which was greater than explicit memory for positive and neutral words) and implicit memory for negative words (which was essentially equivalent for negative, positive, and neutral words).

Consistent with previous research using words, older adults exhibited implicit repetition priming, as measured by superior identification performance on repeated positive and neutral words versus novel words, similar to younger adults (Light et al., 1986; Light and Singh, 1987; Java and Gardiner, 1991; Hultsch et al., 1992; Christensen et al., 1997; Fleischman and Gabrieli, 1998; Davis et al., 2001; Fleischman et al., 2004). These studies did not report controlling the word valence. Our results suggest that the age invariance for implicit memory that has been observed with words in previous research extends to positive and neutral words, but not to negative words. Critically the selective difference for priming for negative words was a memory effect. For baseline words in the test phase that were not seen in the study phase, older and younger adults had similar patterns for performance across valences.

The word-identification paradigm employed in the present study was similar to paradigms used in prior studies of aging and amnesia that have shown a dissociation between implicit and explicit memory. Other word-identification paradigms have yielded different findings (e.g., Abbenhuis et al., 1990; Ward, 2023). Future research may help clarify how these various paradigms differ as to what memory processes are invoked and how they relate to aging.

### 4.3 Theoretical and clinical implications

One plausible explanation for the finding of diminished implicit memory for negative words with aging is implicit emotion regulation. This form of emotional regulation is proposed to occur in the absence of explicit instruction and to be evoked automatically by the stimulus itself (Etkin et al., 2015). Implicit emotion regulation is proposed to drive individuals into “good-for-me” states (e.g., it is better for me to be less negative) and to do this without evoking conscious awareness. This proposed theoretical explanation raises three relevant questions. When would this form of emotion regulation begin, does it influence priming, and is it age invariant?

The results for the positivity effect in attention for visual stimuli suggest that it may be mediated more by automatic than controlled processes. A positivity effect, measured by reaction time in a dot-probe task, appeared from 500 milliseconds of stimulus presentation and did not increase over time (Zsoldos and Hot, 2024). These results are corroborated by other findings reporting positivity effects in attention in older adults at very early stages of sensory processing (Johnson and Whiting, 2013). Attention plays a role in all forms of memory (Stone et al., 2000). Because implicit memory is characterized by automatic processing (Schacter, 1987, 1992), the implicit memory MPE results reported here are consistent with the positivity effects observed at the very early stages of sensory processing.

The explicit memory MPE is based on two theorized mechanisms described as (1) age-related reductions in memory for negative information and/or (2) age-related enhancements in memory for positive information (e.g., Barber and Kim, 2022). The implicit memory MPE in the present study was clearly a reduction of implicit memory for negative words in older adults; implicit memory for positive words was similar in younger and older adults. These two mechanisms may play out differently in age-related changes for explicit memory.

### 4.4 Limitations and future directions

Both limitations and future directions can be considered about this study and its outcome. The results of this study are limited because only two extreme age groups were recruited, younger adults (age range 18–37) and older adults (age range 66–85). We did not examine whether the implicit memory positivity effect would be observed in middle aged adults. Also, the design here required the use of a stimulus category that reliably did not produced the explicit memory positivity effect, words. Whether the implicit memory positivity effect will be observed with stimuli that reliably produce the explicit memory positivity effect such as images related to people, animals, nature, and inanimate objects (Charles et al., 2003) remains unknown. This study recruited younger and older participants from community centers and was conducted in isolated areas within those same community centers. Because some of the participants may have known each other, a post-experimental questionnaire regarding whether the participants knew memory was being tested could not be employed. This raises the possibility that some of the participants may have been aware of the nature of the implicit memory task. It is, however, highly unlikely that they were aware of valence distinctions in relation to memory performance.

The research reported here also provides many opportunities for future investigations. First, it would be informative to study the implicit memory MPE across the lifespan and discern its pattern across adult development. Second, it would be interesting to discover the scope and limitations of the implicit memory MPE across other kinds of stimuli (e.g., faces and pictures) that either have or have not exhibited an explicit memory MPE. Third, there have been neuropsychological dissociations among different kinds of priming with words (Gabrieli et al., 1994), and future research will be required to determine if the implicit memory MPE reported here extends from perceptual priming to more lexical or conceptual forms of priming. Future research may also examine the brain bases of these findings via functional neuroimaging as has been studied in younger and older adults for the response to positive and negative emotional pictures (Mather et al., 2004) by incorporating both explicit and implicit memory measures.

Finally, there may be clinical utility for the findings reported here when considering the diminished reporting of disease-related symptoms (e.g., negative affect) that are non-debilitating (mild to moderate intensity) in older relative to younger patients observed for oncology-related treatments (Cataldo et al., 2013; Johannessen et al., 2023; Morse et al., 2024), pneumonia (Metlay et al., 1997), tuberculosis (Korzeniewska-Kosela et al., 1994; Table 1), depression (Gallo et al., 1994; Lyness et al., 1995), and hearing loss (Wiley et al., 2000; Dillard et al., 2024). To date, age-related differences in the symptom experience have not been explained. Based on the data presented here, a relevant question would be whether there is a relation between diminished symptom reporting and decreased implicit memory for negative information in older adults?

## 5 Conclusion

The tendency to disproportionally remember positive versus negative information in older adults (the MPE) has been based largely on studies using explicit memory measures (e.g., Mather and Knight, 2005; Thomas and Hasher, 2006; Talmi et al., 2007; Kensinger, 2008; Petrican et al., 2008). The present study reveals that a version of the MPE extends to implicit memory in older adults. Future studies may investigate the brain bases of this MPE, and how it may or may not extend to other forms of implicit memory.

## Data Availability

The datasets presented in this study can be found in online repositories. The names of the repository/repositories and accession number(s) can be found below: The datasets [generated/analyzed] for this study can be found at the American Psychological Associations’ Repository on the Open Science Framework [https://osf.io/q2wzb/].

## Footnotes

1 Cedrus Corporation (1991/2023) cedrus. Available at: https://www.cedrus.com
