# Supplementary material for: Implicit memory reduced selectively for negative words with aging
Source: Front Aging Neurosci. 2024 Oct 9;16:1454867. doi: 10.3389/fnagi.2024.1454867 (PMC11497464; doi:10.3389/fnagi.2024.1454867)
Supplement: Supplementary file 1 [file Data_Sheet_1.zip › Table 1.docx]

**Supplementary Table 1. Words for study lists A, B, C and D.**

| **Positive** | | | | **Negative** | | | | **Neutral** | | | |
| --- | --- | --- | --- | --- | --- | --- | --- | --- | --- | --- | --- |
| **A** | **B** | **C** | **D** | **A** | **B** | **C** | **D** | **A** | **B** | **C** | **D** |
| aroma | angel | courage | achieve | betray | agony | accident | cannibal | assume | behavior | absolute | apply |
| awesome | cheerful | delight | award | chemo | autopsy | blame | clot | attitude | bureau | adjuster | block |
| beauty | euphoric | easy | beloved | disease | bubonic | broken | coffin | axis | cellar | avoid | central |
| blissful | excited | fabulous | comedy | evil | cancer | bury | coma | drill | ennui | ground | clipping |
| bonus | fairness | honest | create | extinct | criminal | crack | death | emery | excuse | hail | factor |
| cuddle | faithful | humorous | energize | funeral | cyanide | creep | gang | flier | habitual | hole | hollow |
| daddy | fondness | inspire | funny | hang | enemy | defeated | harm | hide | hallway | merger | hour |
| dance | genius | nourish | joyful | herpes | grief | hatred | hijack | mayor | issue | ought | minor |
| friendly | golden | oasis | kindness | jail | intruder | helpless | hopeless | median | manifold | parking | modicum |
| giggle | happy | pleasure | lovable | lymphoma | jerk | leukemia | illness | packet | observer | part | outer |
| joke | healthy | puppy | magical | nausea | killing | lost | insult | phrase | obtain | possess | prone |
| laughter | musical | smile | partner | obesity | missile | massacre | misfire | posture | pile | reporter | sock |
| lullaby | praise | spring | playful | racism | morgue | murderer | monster | probate | retainer | roommate | spread |
| payday | prize | success | positive | stupid | poison | thief | sewage | rule | search | rotate | square |
| peace | relaxing | sunshine | princess | victim | pollute | vandal | sick | shall | senator | rush | stand |
| travel | seashore | talented | prosper | weak | suffer | virus | slap | shudder | shift | site | third |
| wellness | vacation | thankful | save | whiplash | toxicity | wasp | stealing | street | station | suction | umpire |
| wildlife | wisdom | treasure | winner | wreck | worry | widow | upset | truck | steps | tract | unheard |
