# Supplementary material for: Implicit memory reduced selectively for negative words with aging
Source: Front Aging Neurosci. 2024 Oct 9;16:1454867. doi: 10.3389/fnagi.2024.1454867 (PMC11497464; doi:10.3389/fnagi.2024.1454867)
Supplement: Supplementary file 1 [file Data_Sheet_1.zip › Table 2.docx]

| **Supplementary Table 2. Implicit memory (difference scores) by valence-specific words and age group.** | | | | | | | | | | | | |
| --- | --- | --- | --- | --- | --- | --- | --- | --- | --- | --- | --- | --- |
|  |  | Positive | | | Negative | | | Neutral | | | Total | |
| Age | *N* | *M* | *SEM* |  | *M* | *SEM* |  | *M* | *SEM* |  | *M* | *SEM* |
| OA | 24 | 8.08^a,x^ | 2.98 |  | 8.50^a,x^ | 3.31 |  | 8.92^a,x^ | 4.17 |  | 8.50^a^ | 2.34 |
| YA | 24 | 8.42^a,x^ | 2.27 |  | 19.83^b,y^ | 4.50 |  | 8.67^a,x^ | 2.70 |  | 12.31^a^ | 2.45 |
| Total | 48 | 8.25^x^ | 1.85 |  | 14.17^y^ | 2.87 |  | 8.79^xy^ | 2.46 |  |  |  |

OA = Older adult; YA = Younger adult. Significant differences between OA and YA are shown using

superscripts a and b within the valence-specific words and for the totals; significant differences

between the valences are shown using superscripts x and y within OA and YA and for the totals.

Means with different superscripts differ significantly, *p* < 0.001.
