# Supplementary material for: Implicit memory reduced selectively for negative words with aging
Source: Front Aging Neurosci. 2024 Oct 9;16:1454867. doi: 10.3389/fnagi.2024.1454867 (PMC11497464; doi:10.3389/fnagi.2024.1454867)
Supplement: Supplementary file 1 [file Data_Sheet_1.zip › Table 3.docx]

| **Supplementary Table 3. Word identification item types (times in ms: new, old) by age group and valence.** | | | | | | | | | |
| --- | --- | --- | --- | --- | --- | --- | --- | --- | --- |
|  |  |  | New Time | |  | Old Time | |  | Priming Effect |
| Age | Words |  | *M* | *SD* |  | *M* | *SD* |  | *M* |
| OA | Positive |  | 460.25 | 313.41 |  | 452.17 | 317.76 |  | 8.08 |
| YA | Positive |  | 211.25 | 87.26 |  | 202.83 | 86.25 |  | 8.42 |
| OA | Negative |  | 465.83 | 311.64 |  | 457.33 | 315.92 |  | 8.50 |
| YA | Negative |  | 230.21 | 98.36 |  | 210.38 | 88.08 |  | 19.83 |
| OA | Neutral |  | 470.75 | 312.39 |  | 461.84 | 311.77 |  | 8.91 |
| YA | Neutral |  | 224.29 | 95.27 |  | 215.62 | 95.44 |  | 8.67 |

OA = Older adult; YA = Younger adult. Item type post hoc comparisons (new, old in ms) using

paired-sample *t* tests (one-tailed) were computed for each age group. For OA, new items for positive,

negative, and neutral words were significantly greater than old items: positive words, *t* (23) = 2.71,

*p* = 0.05, *d* = 0.55; negative words, *t* (23) = 2.57, *p* = 0.01, *d* = 0.52; neutral words, *t* (23) = 2.14,

*p* = 0.02, *d* = 0.44. For YA, new items for positive, negative, and neutral words were significantly

greater than old items: positive words, *t* (23) = 3.71, *p* < 0.001, *d* = 0.76; negative words, *t* (23) = 4.41,

*p* < 0.001, *d* = 0.90; neutral words, *t* (23) = 3.21, *p* = 0.002, *d* = 0.66.
