# Supplementary material for: Implicit memory reduced selectively for negative words with aging
Source: Front Aging Neurosci. 2024 Oct 9;16:1454867. doi: 10.3389/fnagi.2024.1454867 (PMC11497464; doi:10.3389/fnagi.2024.1454867)
Supplement: Supplementary file 1 [file Data_Sheet_1.zip › Table 4.docx]

| **Supplementary Table 4. Explicit memory (difference scores) by valence-specific words and age group.** | | | | | | | | | | | | |
| --- | --- | --- | --- | --- | --- | --- | --- | --- | --- | --- | --- | --- |
|  |  | Positive | | | Negative | | | Neutral | | | Total | |
| Age | *N* | *M* | *SEM* |  | *M* | *SEM* |  | *M* | *SEM* |  | *M* | *SEM* |
| OA | 24 | 37.27 | 3.13 |  | 45.60 | 4.03 |  | 35.88 | 4.14 |  | 39.58^a^ | 4.25 |
| YA | 24 | 48.15 | 3.24 |  | 62.27 | 3.52 |  | 51.62 | 3.58 |  | 54.01^b^ | 4.25 |
| Total | 48 | 42.71^x^ | 2.36 |  | 53.94^y^ | 2.91 |  | 43.75^x^ | 2.94 |  |  |  |

OA = Older adult; YA = Younger adult. Significant differences between OA and YA are shown

using superscripts a and b; significant differences among the valences are shown using

superscripts x and y. Means with different superscripts differ significantly, *p* < 0.001.
