# Supplementary material for: Implicit memory reduced selectively for negative words with aging
Source: Front Aging Neurosci. 2024 Oct 9;16:1454867. doi: 10.3389/fnagi.2024.1454867 (PMC11497464; doi:10.3389/fnagi.2024.1454867)
Supplement: Supplementary file 1 [file Data_Sheet_1.zip › Table 5.docx]

| **Supplementary Table 5. Recognition item types (percentages: old, new)**  **by age group and valence.** | | | | | | | | | |
| --- | --- | --- | --- | --- | --- | --- | --- | --- | --- |
|  |  |  | % Old (Hits) | |  | % New (FA) | |  | Recognition |
| Age | Words |  | *M* | *SD* |  | *M* | *SD* |  | *M* |
| OA | Positive |  | 55.09 | 18.96 |  | 17.82 | 12.90 |  | 37.27 |
| YA | Positive |  | 60.65 | 17.02 |  | 12.50 | 10.90 |  | 48.15 |
| OA | Negative |  | 57.87 | 17.25 |  | 12.27 | 10.49 |  | 45.60 |
| YA | Negative |  | 70.14 | 16.69 |  | 7.87 | 10.09 |  | 62.27 |
| OA | Neutral |  | 45.14 | 18.70 |  | 9.26 | 12.33 |  | 35.88 |
| YA | Neutral |  | 58.33 | 17.80 |  | 6.71 | 10.49 |  | 51.62 |

OA = Older adult; YA = Younger adult. Item type post hoc comparisons (old, new %) were

made within each age group (one-tailed). For OA, post hoc comparisons using paired-sample *t* tests

revealed that for positive, negative, and neutral words old items were significantly greater than new

items: positive words, *t* (23) = 11.92, *p* < 0.001, *d* = 2.43; negative words, *t* (23) = 11.33, *p* < 0.001,

*d* = 2.31; neutral words, *t* (23) = 8.68, *p* < 0.001, *d* = 1.77. For YA, post hoc comparisons using

paired-sample *t* tests revealed that for positive, negative, and neutral words old items were significantly

greater than new items: positive words, *t* (23) = 14.88, *p* < 0.001, *d* = 3.04; negative words, *t* (23) = 17.68,

*p* < 0.001, *d* = 3.61; neutral words, *t* (23) = 14.43, *p* < 0.001, *d* = 2.95.
