# Supplementary figures and images for: Implicit memory reduced selectively for negative words with aging
Source: Front Aging Neurosci. 2024 Oct 9;16:1454867. doi: 10.3389/fnagi.2024.1454867 (PMC11497464; doi:10.3389/fnagi.2024.1454867)

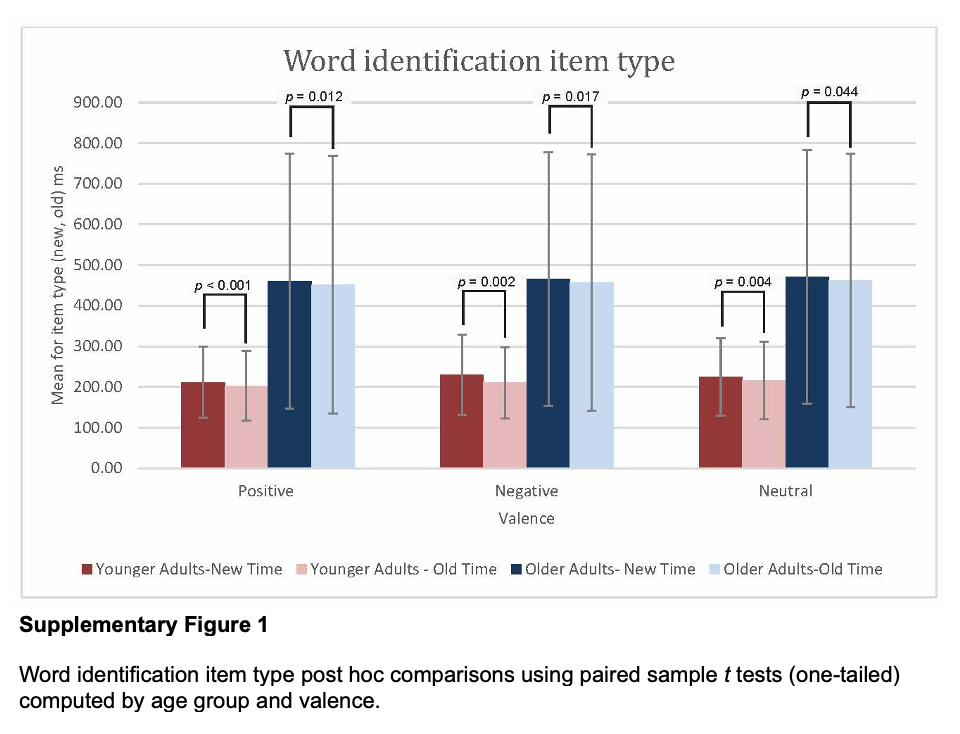

Supplement: Supplementary file 1 [file Data_Sheet_1.zip › Figure 1.jpg]

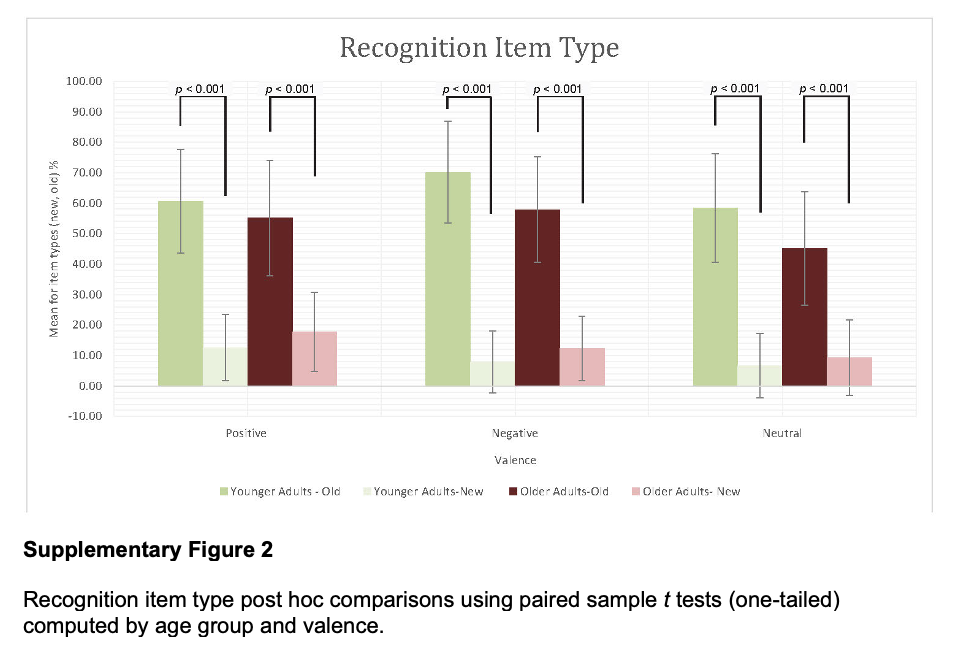

Supplement: Supplementary file 1 [file Data_Sheet_1.zip › Figure 2.jpg]
